# Supplementary material for: A Multi-mRNA Prognostic Signature for Anti-TNFα Therapy Response in Patients with Inflammatory Bowel Disease
Source: Diagnostics (Basel). 2021 Oct 14;11(10):1902. doi: 10.3390/diagnostics11101902 (PMC8534494; doi:10.3390/diagnostics11101902)
Supplement: Supplementary file 1 [file diagnostics-11-01902-s001.zip › diagnostics-1352291 supplementary.pdf]

Supplementary Table S1: Response adjudication definition for the four discovery datasets

Accession	Author	Timepoint of assessment	Clinical Adjudication guidelines
EMTAB7604	Verstockt	6 months after Infliximab and/or adalimumab treatment	In CD patients, response was defined as a Simple Endoscopic Disease (SES-CD) score <= 2. In UC patients, response was defined as Mayo endoscopic sub-score of <= 1 and was considered as endoscopic remission.
GSE14580	Arijs	4 weeks after first Infliximab treatment	Response was defined as complete mucosal healing with a Mayo endoscopic sub-score of 0 or 1 and a grade 0 or 1 on the histological score for ulcerative colitis. Patients who did not achieve healing were considered non-responders although some of them presented endoscopic and/or histological improvement.
GSE16879	Arijs	4 to 6 weeks after first Infliximab treatment	For CDc (CD samples extracted from colon), response was defined as complete mucosal healing with a decrease of at least 3 points on the histological score and decrease to a Mayo endoscopic sub-score of 0 or 1. For CDi (CD samples extracted from Ileum), response was defined as patients with a clear improvement of the ulcerations and a decrease on the histological score were considered responders. Patients who did not achieve this healing were considered non-responders although some of them presented endoscopic and/or histologic improvement.
GSE23597	Toedter	At weeks 8 post Infliximab treatment	Clinical response was defined as a decrease from baseline in the total Mayo score of at least 3 points and at least 30 percent, with an accompanying de- crease in the sub-score for rectal bleeding of at least 1 point or an absolute sub-score for rectal bleeding of 0 or 1.

Supplementary Table S2: Pooled summary statistics for 324 DE genes. Highlighted genes in red constitute the 7-gene signature.
Note: Refer to additional file: Supplementary_Table_2.xlsx

Supplementary Table S3: Test characteristics of each AUROC at its Youden cutoff.

	Accuracy	Sensitivity	Specificity
**E-MTAB-7604**	0.75	0.79	0.72
**GSE14580**	0.92	1.00	0.88
**GSE16879**	0.89	0.90	0.88
**GSE23597**	0.87	0.96	0.57
**Mean**	0.86	0.91	0.76

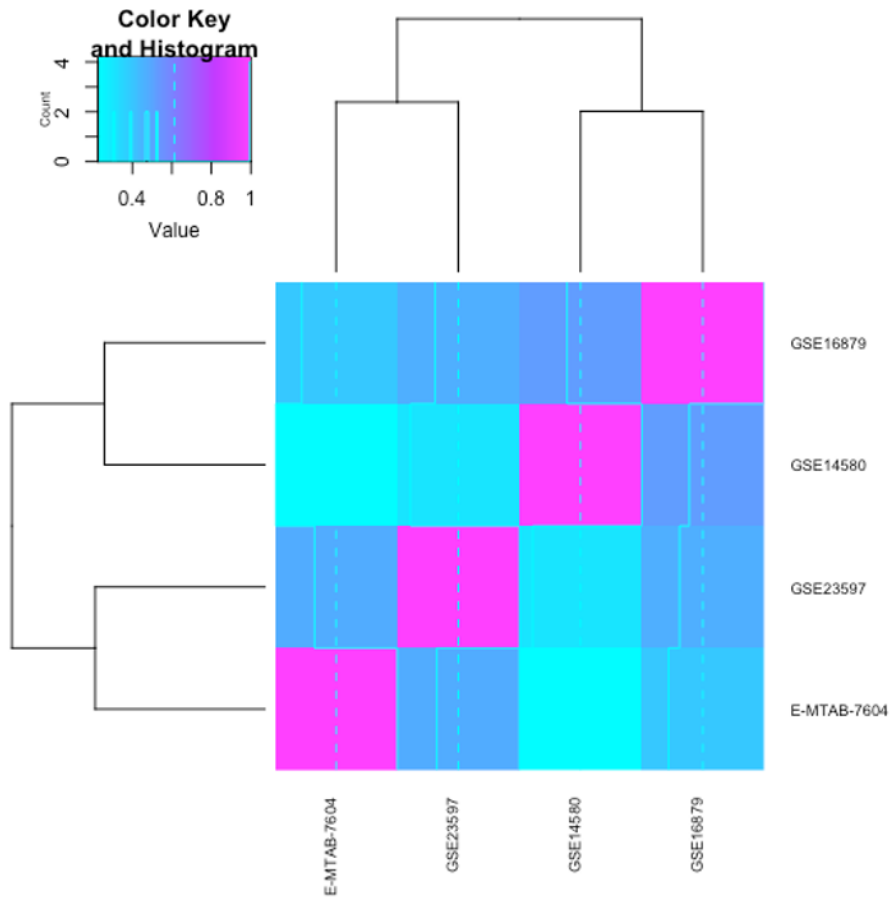

Supplementary Figure S1: Gene effect size correlations across 4 datasets assessing patient response to anti-TNFα therapy. Hierarchical cluster heatmap of all gene effect sizes.
